# The development and validation of the CARe Burn Scale: Child Form: a parent-proxy-reported outcome measure assessing quality of life for children aged 8 years and under living with a burn injury

**DOI:** 10.1007/s11136-020-02627-x

**Published:** 2020-09-09

**Authors:** Catrin Griffiths, Ella Guest, Timothy Pickles, Linda Hollèn, Mariusz Grzeda, Philippa Tollow, Diana Harcourt

**Affiliations:** 1 Centre for Appearance Research (CAR), Department of Health and Social Sciences, University of the West of England, Frenchay Campus, Coldharbour Lane, Bristol, BS16 1QY UK; 2 Centre for Trials Research (CTR), Cardiff University, Cardiff, UK; 3 Centre for Academic Child Health, Bristol Medical School, University of Bristol, Bristol, UK

**Keywords:** Parent, Paediatric, Child, PROM, Burn, Rasch

## Abstract

**Purpose:**

Patient-reported outcome measures (PROMs) identify patient needs and therapeutic progress. This paper outlines the development and validation of the CARe Burn Scale: Child Form, a parent-proxy-reported outcome measure that assesses quality of life in children aged 8 and under living with a burn injury.

**Methods:**

A literature review and interviews with 12 parents of children with a burn and seven health professionals informed the development of a conceptual framework and draft PROM. Cognitive debriefing interviews with 18 parents and eight health professionals provided feedback to ascertain content validity, and 311 parents took part in field testing. Rasch and traditional psychometric analyses were conducted to create a shortened version. Further psychometric analyses with 133 parents tested the shortened CARe Burn Scale in relation to other parent-proxy measures.

**Results:**

The final conceptual framework included 5 domains: *Social and Emotional Difficulties, Social and Emotional Well-Being, Wound/Scar Discomfort, Wound/Scar Treatment* and *Physical Abilities*. Two scales fulfilled Rasch and traditional psychometric analyses, providing evidence of construct validity, acceptability, and reliability. Three scales did not fulfil the Rasch criteria and were retained as checklists. Compared to other parent-proxy measures, individual CARe Burn Scales correlated moderately with similar constructs and had low correlations with dissimilar constructs, indicating evidence of criterion validity (concurrent and discriminant).

**Conclusions:**

The CARe Burn Scale: Child Form can be used to measure children’s quality of life after having a burn injury which can inform rehabilitation and surgical decision-making.

**Electronic supplementary material:**

The online version of this article (10.1007/s11136-020-02627-x) contains supplementary material, which is available to authorized users.

## Introduction

Burn injuries are the fourth most common cause of trauma in the world, with 11 million people requiring medical intervention including surgery each year [1]. Children under 5 years of age are at the greatest risk of burn injuries as they physically develop and become increasingly active, impulsive, and curious, but lack awareness [2, 3].

Scarring and physical impairment are common with burns [4]. Irrespective of a child’s age, a burn can have a significant psychosocial impact, both for the child and family members [5]. Many undergo numerous surgical and rehabilitation interventions to improve wound healing and scarring, which can be physically and emotionally challenging [6]. Children can struggle with coming to terms with the traumatic event, while changes in physical appearance, pain, restricted physical abilities and adherence to treatments such as dressing changes can be hard to cope with [7–9]. Collecting and effectively using health-related patient-reported outcome data (PROM) are vital during surgical and rehabilitation decision-making in burn care since it pin-points how burn scars impact health and, consequently, how intervention may improve these outcomes. Yet the UK’s National Burn Care Review (2001) [10] highlighted the lack of systematic assessment of burn patients’ experiences of their treatment, scarring and psychosocial functioning, and emphasised that the development of new PROMs for this population was vital to support decision-making regarding care, including rehabilitation and reconstructive surgery. Parent-proxy measures are deemed most appropriate to capture young children’s outcomes after burn injuries, since children under the age of 8 are not considered to have the skills needed to express complex concepts such as thoughts and feelings and to answer PROMs reliably [11].

Some validated parent/caregiver-proxy measures specifically assessing quality of life and health outcomes for children aged 8 and under with a burn are available [12]. These include the Children’s Burns Outcome Questionnaire for children aged 5 years and under (CBOQ) [13] and the Brisbane Burn Scar Impact Profile (BBSIP) for caregivers of children aged less than 8 years [14]. However, there are certain aspects relating to a child’s health when living with a burn that are not included in existing PROMs. For example, their questions are not specific to burn scars [13], they do not include the positive emotional well-being of a child living with a burn [13, 14] or do not include the wound stage of a burn injury [13, 14], which is essential information for treatment decision-making and care. The development of parent-proxy measures remains a priority in order to assess children’s needs at any stage of burn recovery in order to inform surgical and rehabilitation decision-making [15].

This paper outlines the development and validation of the CARe Burn Scale: Child Form, a burn-specific parent/carer-proxy reported outcome measure to assess quality of life for children aged 8 and under who have had a burn.

## Methods

The CARe Burn Scale: Child Form was developed following an established three-stage development and validation process [16–19], considered the gold standard for developing and evaluating PROMs. This involved item generation (developing a conceptual framework using a literature review, qualitative interviews with patients and expert opinion), item reduction (using psychometric criteria such as Rasch analysis), and psychometric evaluation.

Ethical approvals were obtained from the University of the West of England and NHS Research Ethics Committees. Informed consent was obtained from all individual participants included in the study.

### Stage 1: Conceptual framework development

Semi-structured interviews were conducted with parents or carers of children aged 8 and under who had experienced a burn and burn-specialist health professionals to explore, in-depth, the impact of a burn on a child’s health and well-being. When this paper refers to parents, this also includes carers who are not the child’s parent but are still legally responsible for caring for the child such as foster parents or other family members. Face-to-face/telephone interviews were conducted between April 2013 and October 2013, recorded, transcribed verbatim and subjected to a thematic analysis [20]. A conceptual framework developed from these findings outlined the key aspects of a child’s well-being when living with a burn.

### Stage 1.2: Item generation, initial scale formation, and pre-testing

Using the health professional and parent/carer interview data, an extensive list of items was created for each conceptual framework domain, using parents’ own words or phrases to increase the content validity of the items. A literature review of PROMs used in paediatric burn care was conducted, relevant parent-proxy measures were obtained, reviewed, and items included in these scales that were not discussed in the interviews were added to the measure. Psychosocial specialists from UK burn services provided feedback on the measure to ensure it was comprehensive.

Cognitive debriefing interviews [16, 19] were conducted with parents who reviewed the scale and gave feedback on their understanding of the items and the response categories, and they suggest new items to ensure that the scale had good face validity and was relevant and understandable to parents [21].

### Stage 2: Item reduction

The measure was field-tested in 11 NHS Burn Services with parents of children aged 8 and under who had sustained a burn injury (of any size and location on their body) and could read English sufficiently well to complete the questionnaire. Eligible participants were invited to take part while attending hospital clinics or questionnaires were mailed to them. Questionnaires could be completed on paper or online, via a secure survey link (www.qualtrics.com).

#### Rasch measurement psychometric analysis

Item reduction was informed by the Rasch measurement model/analyses [22–27] using RUMM2030 [28] based on the following criteria which the authors have previously reported in the development of a burn-specific PROM for adults with a burn injury [29].

#### Item fit statistics

Rasch analysis tests whether the observed data are consistent with the responses predicted by the Rasch model. Two indicators were investigated: (1) item–trait interaction (a non-significant (*p* value > 0.05) chi-square value would indicate negligible deviation between the collected data and expectations of the model); (2) the residual for each item in the range of − 2.5 to + 2.5 and non-significant chi-square values (Bonferroni-adjusted significance level of 0.01) indicates good fit.

#### Person separation index (PSI)

The PSI assesses whether the measurement of participants is reliably separated. A value of 0.7 or higher suggests the possibility to distinguish at least two groups of patients [30, 31].

#### Local dependency

A residual correlation > 0.3 above the mean residual correlation (of all item pairs for that scale) shows a problem with fit [32].

#### Unidimensionality

Smith’s procedure [33] was used to measure unidimensionality within individual scales. This identifies if the person estimates derived from the most diverse subsets of items are significantly different. If the proportion, or the lower bound of the 95% confidence interval of significant (*p* < 0.05) *t* tests, is less than 5% it indicates unidimensionality.

#### Differential Item Functioning

Differential Item Functioning analysis (DIF) [34] assesses whether item parameters remain invariant across different groups of patients. Item difficulties were compared across the following: age (split based on median: 0–2 vs 3–8 years), gender (male vs female), burn type (burn wound vs burn scar only vs no wound/scar), and body part affected (‘hands, bottom, upper legs, lower legs, feet’ vs ‘head/face, neck, chest, back, lower arms, upper arms). This analysis investigated the issue of possible bias from misfit of the data to model. Uniform and non-uniform DIF were investigated graphically (inspection of item characteristic curves (ICCs) for different groups) and by results of analysis of variance (Bonferroni-adjusted significance level of 0.05).

#### Targeting and item locations

Graphical comparisons of the item distributions and person locations determined whether they covered more or less the same areas of the Rasch continuum. Large floor and ceiling effects indicates the inability to differentiate between participants along the construct [35].

#### Item thresholds

For each item, the use of response categories scored with successive integer scores indicated a continuum of increasing impact. This assumption was tested by ordering the thresholds specified by the Rasch analysis.

#### Traditional psychometric analysis (Classical Test Theory)

Traditional psychometric analysis using Classical Test Theory (CTT) was conducted using SPSS Statistics 23 [36] to show how the scale operates based on the CTT criteria: Cronbach’s alphas and item–total correlations.

### Stage 3: Further psychometric evaluation

Using the same recruitment methods and criteria as in Stage 2, the final scale was tested in comparison to other parent-proxy measures to ascertain evidence of criterion validity (concurrent and discriminant) [17]. Participants who had previously taken part in Stage 2 were also invited to take part. Stata v.15.1 [37] was used to test data quality, scaling assumptions, and validity and reliability:

- *Criterion validity*: The individual scales from the CARe Burn Scale: Child Form were compared with existing parent-proxy measures of similar constructs (Paediatric Quality of Life Inventory (PedsQL) [38] and the Patient and Observer Scar Scale (POSAS-Observer Form) [39], which have previously used in burn care and research. The CARe Burn Scales were hypothesised to have moderate/high correlations with related constructs and low/no correlations with dissimilar constructs. Criteria were used as guides in terms of the magnitude of correlations, rather than pass/fail benchmarks (high correlation, *r* < 0.70; and moderate correlation, *r* < 0.30 to 0.70).

It was hypothesised that:

- *Wound/Scar Discomfort*, *Wound/Scar Treatment, Physical Difficulties*, and *Physical Abilities* would moderately correlate with the PedsQL Physical Functioning subscale.
- *Social Situations* and *Avoidance Behaviours* would moderately correlate with the PedsQL Social Functioning subscale.
- *Social and Emotional Difficulties* and *Social and Emotional Well-being* would moderate correlate with the PedsQL Social Functioning and Emotional Functioning subscales.
- *Trauma Symptoms* would moderately correlate with the PedsQL Emotional Functioning subscale.
- *Nursery/School* would moderately correlate with the PedsQL School Functioning subscale.
- *Child’s Wound/Scar Dissatisfaction* and *Parent Dissatisfaction with the Appearance of Wounds/Scars* would moderately correlate with the POSAS.

Traditional psychometric measurement properties were also examined: acceptability [percentage of missing data (< 10% acceptable)], internal consistency (Cronbach’s alpha (> 0.70 acceptable)), and acceptable item–total correlations (> 0.30).

The relationships between CARe Burn Scale subscales and sociodemographic and burn-related variables (age, gender of child and parent, ethnicity, marital status, cause of burn and time since burn) were examined using regression analyses to determine the extent to which scores were influenced by these variables.

## Results

### Stage 1.1: Conceptual framework formation

Twelve parents (9 female, 80% White ethnicity, see Table 1) and seven clinical psychologists who worked in paediatric burn care were interviewed (analysis of these interviews is reported in full in Guest et al., [15] and Griffiths et al. [40]). Thematic analysis identified a range of themes. Thirteen key domains formed the initial conceptual framework reflecting a child’s quality of life after a burn:

1. *Wound/Scar Discomfort*
2. *Physical Difficulties*
3. *Physical Abilities*
4. *Physical Development*
5. *Wound/Scar Treatment*
6. *Social Situations*
7. *Avoidance Behaviours*
8. *Social and Emotional Difficulties*
9. *Social and Emotional Well-Being*
10. *Child’s Wound/Scar Dissatisfaction*
11. *Parent Dissatisfaction with the Appearance of Wounds/Scars*
12. *Trauma Symptoms*
13. *Nursery/School*

**Table 1:** Stage 1: Qualitative interview study participant demographics

*N*	Age	Ethnicity	Cause of burn	Body parts affected by burn	Age child was injured	Child’s age now	Time since burn injury
P1	34	Asian	Flame	Arms, legs, face, neck, back	2.5 years	8 years old	5.5 years
P2	40	White	Flame	Legs	5 years old	7 years old	1 year and 9 months
P3	45	White	Flame	Legs	5 years old	7 years old	1 year and 9 months
P4	52	White	Flame	Chest, arms, back and neck	3 years old	5 years old	13 months
P5	53	White	Flame	Chest, arms, back and neck	3 years old	5 years old	13 months
P6	37	White	Liquid	Face, neck and chest	5 years old	8 years old	3 years
P7	28	White	Flame	Chest, stomach, neck, face, arms	5 years old	8 years old	3 years
P8	28	White	Flame	Chest, stomach, neck, face, arms	5 years old	8 years old	3 years
P9	39	White	Liquid	Chest, face, arms, legs and feet	1.5 years old	12 years old	10 years
P10	40	White	Flame	Face, ear, shoulder and back	11 years old	12 years old	12 months
P11	38	Black	Contact	Left leg	6 months old	11 years old	10 years and 6 months
P12	42	Black	Contact	Arm and hand	6 months old	11 years old	10 years and 6 months

*N* = participant number

### Stage 1.2: Item generation, initial scale formation, and pre-testing

Initially, 121 items were created. Cognitive debriefing interviews were conducted with 18 parents of children aged 0–8 with a burn (83% female, mean parent age: 41:19, SD: 6.34, mean child age when injured: 3.91, SD: 2.84), and feedback was sought from eight health professionals (six clinical psychologists, one play specialist, one health psychologist) resulting in minor edits (changes to item wording, providing more burn-specific examples) and a further 29 items being added to the existing domains.

Health professionals reviewed and provided feedback on the scale and changes were made in light of their responses. Parents then reviewed the amended scale, which was edited after each interview in response to the feedback, to ensure that subsequent parents were reviewing the most recent version. A total of 150 items were then field-tested.

### Stage 2: Field testing, item reduction and Rasch analysis

#### Sample

A total of 311 participants completed the CARe Burn Scale: Child Form. Table 2 provides the participant characteristics. The largely supported rule of thumb is that in order to perform an accurate and precise Rasch analysis with over 99% confidence and item calibrations within ± 0.5 logits, a sample size of 250 is needed [41].

**Table 2:** Stage 2: Study patient characteristics (*N* = 311)

Demographics			
		*N*	%
Child’s age in years when injured	*M* = 2.0 (SD: 1.84)	307	
Child’s gender	Male	173	55.6
	Female	135	43.4
Injury status	Burn wound	14	4.5
	Burn scar	172	55.3
	Both wound and scar	24	7.7
	No wound scar	94	30.2
Body part affected	Head or face	89	28.6
	Neck	71	22.8
	Chest	117	37.6
	Back	28	9.0
	Lower arms	60	19.3
	Upper arms	84	27.0
	Hands	114	36.7
	Bottom	10	3.2
	Upper legs	37	11.9
	Lower legs	23	7.4
	Feet	34	10.9
Parent gender	Male	43	13.6
	Female	262	82.6
	Missing	12	3.8

Percentages in the above table may not sum to 100% as they show the share of given group in the whole sample of 311 burn patients

#### Item reduction and scale formation

The original scale field-tested had 150 items. During the item reduction stage, 124 items were dropped, which resulted in 26 items in the final scale. Rasch analysis identified a solution for three out of the 13 scales (*Parent Dissatisfaction with the Appearance of Wounds/Scars*, *Social and Emotional Difficulties* and *Social and Emotional Well-being*).

However, based on reviewer feedback of this manuscript, *Parent Dissatisfaction with the Appearance of Wounds/Scars* was deemed to not reflect a true parent-proxy scale since it involved parents reporting their own feelings about their child’s scarring, rather than reporting on their child’s health after a burn. This subscale was therefore removed, which resulted in the final measure having two scales where as Rasch solution was found.

Seven of the 10 scales where a Rasch solution was not found were dropped (Physical Difficulties, Physical Development, Social Situations, Avoidance Behaviours, Child’s Wound/Scar Dissatisfaction, Trauma Symptoms and Nursery/School). Three (Wound/Scar Discomfort, Physical Abilities, Wound/Scar Treatment) were retained based on theoretical insight via communication with burns clinicians who reported during the item reduction stage that these scales would be still useful in their clinical practice. For further information about the checklists, please see below.

The final scale has 26 items and 5 scales: S*ocial and Emotional Difficulties*, *Social and Emotional Well-being*, *Wound/Scar Discomfort, Physical Abilities, and Wound/Scar Treatment.*

#### Rasch measurement psychometric analysis

In the final version of the CARe Burn Scale: Child Form, a Rasch solution was identified for two scales (Table 3): *Social and Emotional Difficulties* and *Social and Emotional Well-being*. 25 items across those two scales were reduced to 15 based on item reduction implemented via Rasch analyses. Scale reliability was generally supported by high PSI. Fit to the Rasch model was good, with all item–trait interactions non-significant and no items with fit residuals out of range or presenting significant *Χ*^2^ values. All final scale solutions contain no items with reversed thresholds. One *Social and Emotional Difficulties* item displayed non-uniform DIF for age, but its inclusion allowed for overall fit to the Rasch model and higher reliability, so the decision was made to keep it. The vast majority of items did not exhibit DIF, indicating that items remain invariant across different groups of patients. However, both solutions required response thresholds to be collapsed for this to be the case. For *Social and Emotional Difficulties*, the third, fourth, and fifth categories (Some of the time, Most of the time and All of the time) were collapsed, and for *Social and Emotional Well-being*, the first, second, and third categories (None of the time, A little of the time, Some of the time) were collapsed. All pairs of items within each scale had a residual correlation less than 0.3 above the mean (of all item pairs for that scale), supporting local independence among items. Smith’s procedure [33] confirmed unidimensionality for all three scales.

**Table 3:** Stage 2 study: summary of CARe Burn Scale: Child Form psychometric analyses

CARe Burn Scale	Rasch analyses											Traditional psychometric analyses	
	Number of items retained in final version	Item–trait interaction* Χ*^2^;df;*p*	Person separation index	Number of items with fit statistics residuals outside the band of − 2.5, + 2.5	Items with significant * Χ*^2^ value *	Number of items with thresholds reversed	Number of pairs of items with residual correlation > 0.3 above the average	Number of items with DIF****	Unidimensionality tests: Highest eigenvalue** (proportion of significant *t *tests***)	Item locations Logits (range of items) [range of category thresholds]	Person location logits (mean, SD) and Person Fit Residual [mean, SD]	Cronbach’s alpha	Item–total correlation Mean (range)
Social and Emotional Difficulties	11	59.1;44;0.06	0.787	0	0	0	0	0	1.780 (6%, 95% CI LB 3%)	(− 1.364, d1.877) [− 1.893, 2.944]	(1.519, 1.743) [− 0.316, 1.061]	0.894	0.636 (0.545–0.731)
Social and Emotional Well-being	4	11.7;12;0.468	0.784	0	0	0	0	1	1.564 (1%)	(− 0.937,0.773) [− 3.434, 4.980]	(1.377, 2.892) [− 0.460, 0.682]	0.864	0.719 (0.697–0.761)

*Baseline significance level was 0.01, which was after adjusted with Bonferroni correction for number of items

**Criterion of unidimensionality assumed was that highest eigenvalue for matrix of residual correlations should be < 2.0

***Criterion of unidimensionality acceptance is the proportion, or lower bound of 95% confidence interval (95% CI LB), is under 5%

****Baseline significance level 0.05 Bonferroni-adjusted

Despite finding two solutions, all scales had gaps in the person location and item threshold distributions, meaning that it is not possible to wholly reflect the range of the continuum, and ceiling effects in their person distributions. The results showed that *Social and Emotional Well-being* had one item with non-uniform DIF on age, though the evidence for these DIF issues is weak (*p* value just less than the α = 0.05 Bonferroni-corrected level) but are reported for full disclosure. For *Wound/Scar Discomfort*, an item displayed DIF on burn type, plus there was poor reliability, a ceiling effect, and overall misfit. For *Physical Difficulties*, multiple items had multiple issues with combinations of model fit and DIF on gender and burn type and body part affected, with poor reliability, a ceiling effect, and overall misfit. For *Physical Abilities* and *Wound/Scar Treatment*, there was poor reliability and a ceiling effect. For *Physical Development*, several items had multiple issues with combinations of model fit and local independence, with poor reliability, a ceiling effect, and overall misfit. For *Social Situations*, there was poor reliability, a ceiling effect, and overall misfit. For *Avoidance Behaviours*, several items had multiple issues with combinations of model fit, local dependence, and DIF on age, with poor reliability and a ceiling effect. For *Child’s Wound/Scar Dissatisfaction*, an item displayed DIF on burn type, plus there was poor reliability and a ceiling effect. For *Trauma Symptoms*, no model would converge given the items and data within them. For *Nursery/School*, multiple items had multiple issues with model fit, plus there was poor reliability and a ceiling effect.

#### Traditional psychometric analyses (Classical Test Theory)

All scales with Rasch solutions passed criteria for reliability (Cronbach’s alpha > 0.80) and validity (all item–total correlation coefficients > 0.30) (Table 3).

#### Checklists

Seven of the 10 scales that did not meet Rasch criteria were removed from the PROM and three were retained based on theoretical insight. During the analysis stage, we communicated with health professionals involved in the pre-testing study and they recommended retaining the *Wound/Scar Treatment*, *Wound/Scar Treatment*, and *Physical Abilities* scales as they would be still useful in their clinical practice.

The checklists are scored by summing all items within them. They can be used by clinicians and researchers to ascertain further information about the domain being examined. However, since they are not psychometrically valid, they are not intended for use in psychometric analysis. Within the three retained scales, ‘Not at all’ was the most commonly endorsed response category for items in *Wound/Scar Treatment*, while ‘Not at all’ and ‘All of the time,’ respectively, were the most commonly endorsed categories in the *Wound/Scar Discomfort* and *Physical Abilities* scales (Table 4).

**Table 4:** Stage 2 study: Number (%) of participants to endorse each response category of the symptom checklist

Scale/item	Not at all		A little		Somewhat		Quite a bit		A lot		N/A	
	*N*	%	*N*	%	*N*	%	*N*	%	*N*	%	*N*	%
*Wound/Scar Care and Treatments*												
1…during wound dressing or bandage changes	120	38.6	17	5.5	5	1.6	10	3.2	15	4.8	131	42.1
2…when washing the body part affected by their burn wounds/scars	204	65.6	36	11.6	8	2.6	9	2.9	15	4.8	35	11.3
3…when wearing bandages, dressing or pressure garments (e.g. tight fitted clothing that helps control scarring)	131	42.1	21	6.8	17	5.5	4	1.3	7	2.3	119	38.3
4…during creaming or massage	160	51.4	57	18.3	20	6.4	11	3.5	13	4.2	45	14.5
5..when completing physiotherapy exercises such as stretching	136	43.7	16	5.1	2	0.6	1	0.3	5	1.6	138	44.4
6…when taking medication for their burn	131	42.1	7	2.3	3	1.0	4	1.3	5	1.6	143	46.0

	None of the time		A little of the time		Some of the time		Most of the time		All of the time		N/A	
	*N*	%	*N*	%	*N*	%	*N*	%	*N*	%	*N*	%
*Physical Abilities*												
1…do the physical activities that they wanted to do (e.g. walk/run/sit down/play)	36	11.6	3	1.0	4	1.3	5	1.6	227	73.0	29	9.3
2…do the physical activities that other children their age/ability can do	29	9.3	2	0.6	7	2.3	16	5.1	238	76.5	15	4.8
3…have enough energy to do the activities they want to do	28	9.0	1	0.3	4	1.3	11	3.5	248	79.7	14	4.5

	Never		Sometimes		Often		Most of the time		Always		N/A	
	*N*	%	*N*	%	*N*	%	*N*	%	*N*	%	*N*	%
*Wound/Scar Discomfort*												
1…itchy	186	59.8	62	19.9	20	6.4	10	3.2	6	1.9	19	6.1
2…painful	233	74.9	37	11.9	6	1.9	5	1.6	5	1.6	17	5.5

### Stage 3: Further psychometric evaluation

#### Sample

133 parents took part in further psychometric testing part (19 men, 105 women representing 55 girls and 67 boys, gender not provided for 11 parents and children). Child’s mean age when injured was 4.0 years (SD: 2.1) (see Table 5).

**Table 5:** Stage 3: Patient characteristics study 2 (N total = 133)

Characteristics	Value	*N*
*Child’s age in years now* (Mean, SD)	4.0 (2.1)	124
*Child’s age in years at time of injury* (Mean, SD)	2.5 (1.5)	121
*Time in years since burn*		
Mean (SD)	1.6 (1.5)	121
Range	0–7	
*Child’s gender*		
Male	67 (54.9%)	122
Female	55 (45.1%)	
*Parent gender*		
Male	19 (15%)	133
Female	114 (85%)	
*Parental age*		
Mean (SD)	35.2 (6.3)	105
Range	21.0 – 63.8	
*Parental ethnicity*		123
White British	97 (78.9%)	
White other	11 (8.9%)	
Indian	4 (3.3%)	
Other	4 (3.2%)	
Pakistani	2 (1.6%)	
Chinese	2 (1.6%)	
Black/Black British: Caribbean	1 (0.8%)	
Black African	1 (0.8%)	
Mixed	1 (0.8%)	
*Parental marital status*		124
Married	83 (66.9%)	
Civil partnership	3 (2.4%)	
Single, never married	16 (12.9%)	
Separated	4 (3.2%)	
Divorced	0 (0.0%)	
Cohabiting	16 (12.9%)	
In relationship but not living together	2 (1.6%)	
*Cause of burn*		125
Flame	1 (0.8%)	
Liquid	76 (60.8%)	
Contact	39 (31.2%)	
Electricity	0 (0.0%)	
Chemical/acid	1 (0.8%)	
Other	8 (6%)	
Body part burnt*		133
Head	39 (29.3%)	
Neck	28 (21.0%)	
Chest	42 (31.6%)	
Back	11 (8.3%)	
Lower arms	26 (19.6%)	
Upper arms	30 (22.6%)	
Hands	52 (39.1%)	
Bottom	3 (2.3%)	
Upper legs	15 (11.3%)	
Lower legs	12 (9.0%)	
Feet	17 (12.8%)	

*Does not add up to 100% since participants could have one or more body parts burnt. *N* = number of participants

#### Traditional psychometric analyses

All scales exceeded criteria for validity and reliability (Table 6 and Appendix A—Supplementary Material). Internal consistency was supported by high Cronbach’s alpha coefficients (> 0.85). Item–total correlations (range of means, 0.53 to 0.75) were also high. Missing data were slightly higher than 10% for all items (range 14%–16%). A comparison of the results with and without missing data showed that the difference in Cronbach’s alphas was negligible (Appendix B—Supplementary Material).

**Table 6:** Stage 3: Traditional psychometric analyses

Traditional psychometric analyses—reliability			
Scales	*N* items	Cronbach's alpha	CITC (mean, range)
Social and emotional difficulties	11	0.86	0.53, 0.27–0.71
Social and emotional well-being	4	0.86	0.71, 0.67–0.75

*CITC* Corrected Item–Total Correlation, only provided for scales with > 2 subscales

The individual subscales in the CARe Burn Scale: Child Form and the other parent-proxy measures were correlated providing evidence of criterion validity (concurrent) (Appendix C—Supplementary Material). The CARe Burn Scales correlated moderately with most of the PedsQL subscales. In particular, *Social and Emotional Difficulties* correlated moderately with all of the PedsQL subscales. *Social and Emotional Well-Being* was moderately correlated with the PedsQL Social and Psychosocial Health subscales.

As predicted, *Social and Emotional Well-being* had low correlations with the PedsQL Physical Functioning subscale, which provides evidence of discriminant validity. An unexpected moderate correlation was identified between *Social and Emotional Difficulties* and the PedsQL Physical Functioning subscale.

Regression analysis identified significant relationships between two of the individual CARe Burn Scales and sociodemographic variables (Appendix D—Supplementary Material). Older age and shorter time since burn were both associated with more social and emotional difficulties. Parents of girls were more likely to report that their child had higher levels of social and emotional well-being. However, since the majority of regression coefficients (15 out of 18) were non-significant, this provides evidence of discriminant validity.

## Discussion

Parents played a vital role in the development of this new PROM, informing item generation and reviewing draft versions of the scale. The final domains and items included in the CARe Burn Scale: Child Form have therefore been verified by parents as important to a child’s health and well-being after experiencing a burn injury. The CARe Burn Scale: Child Form is an important addition to the very few existing parent-proxy measures of a child’s health after a burn, since it is the first burn-specific PROM to include both the wound and scar stages of recovery and to include the positive aspects of a child’s social and emotional health when living with a burn.

Parent reports are vital when investigating quality of life for children who are still too young to reliably report complex issues such as their thoughts and feelings; however, they must be treated as a different perspective to the child’s own. Most children over the age of 8 with a burn can reliably report their own health using PROMs developed for their age group, such as the CARe Burn Scale: Young Person Form [42].

During the item reduction stage, the number of domains in the scale was reduced substantially from 13 to 5. All of the original domains were highlighted by parents/caregivers as important to a child’s health when living with a burn injury during the item generation stage (i.e. in the qualitative interviews and cognitive debriefing interviews) and, ideally, all domains would be retained. However, the rationale for developing this parent-proxy was to develop a robust measure of health outcomes for children who are living with burn injuries, so it was imperative for the scales to work psychometrically. When a scale is psychometrically robust, it increases the chances that the scale will be able to identify clinical changes [22]. Our aim was also to produce a measure which was as short as possible for practical use in busy burns clinics, focussed on domains that are valid for the majority of parents/caregivers and are likely to detect clinical changes. We followed the three-stage PROM development and validation process [18], which recommends using Rasch analysis for item reduction, which identified only the scales that were psychometrically robust. Although a limitation of using this technique is that the final scale does not cover the full scope of quality of life contained within the original scale, three additional checklists focussing on physical health were retained to rebalance the breadth of the scale. Taken together, the subscales and checklists provide a valuable insight into the issues deemed important for a child’s health when living with a burn reported in this and previous studies [12].

Although the checklists are not intended to be used in psychometric analysis, they can be used by clinicians to gain more information about a child’s health. This is particularly important during treatment planning. Also the two psychological and behavioural scales that were retained based on the Rasch analysis (Social and Emotional Well-being and Social and Emotional Difficulties) are likely to pick up the psychological and social disturbances and adjustment related to physical health problems such as wound/scar pain/discomfort and physical health challenges that children often experience [2, 7, 12].

Other existing parent-proxy measures of a child’s health after a burn injury such as the CBOQ [13] and the BBSIP [14] do include the negative emotional impact on the children. However, the CARe Burn Scale – Child Form, is the only scale to include a domain to measure the positive side of a child’s social and emotional health (*Social and Emotional Well-being)* in addition to the negative social and emotional impact *(Social and Emotional Difficulties).* Measuring both the negative and positive social and emotional impact is essential in order to gain a comprehensive assessment of the long-term impact on a child. The CARe Burn Scale – Child Form offers clinicians and researchers an additional tool to evaluate the impact of children’s burns and treatment effectiveness.

## Limitations

Although the regression analysis indicated that the gender or ethnicity of parents did not significantly influence the results, the sample was still relatively homogeneous in terms of gender and ethnicity, with most participants being White females. This limits the generalizability of the results. Future research should include more ethnically diverse samples and men.

Further ongoing validation work is still required. Specifically, test–retest reliability and responsiveness data are needed to explore the reliability of the scale and its ability to detect clinical changes over time. This is necessary in order that suitably validated PROMs are available to enable burn care teams to measure the effectiveness of treatment and surgery from a parent’s perspective.

The scale described in this paper is only valid for measuring outcomes for children aged 8 and under with a burn, but it is part of a suite of burn-specific PROMs developed by the authors that assess outcomes of young people aged 8–17 years [42], adults [29], and parents who are supporting a child with a burn [43].

## Conclusions

The CARe Burn Scale: Child Form is now available for health professionals and researchers use to identify the needs of children aged 8 years and under following a burn injury. Visit www.careburnscales.org.uk to access the full set of CARe Burn Scales.

## Electronic supplementary material

Below is the link to the electronic supplementary material.

Supplementary file1 (DOCX 22 kb)

Supplementary file2 (DOCX 18 kb)

Supplementary file3 (DOCX 19 kb)

Supplementary file4 (DOCX 26 kb)

Supplementary file5 (DOCX 26 kb)
