# Supplementary material for: The development and validation of the CARe Burn Scale: Child Form: a parent-proxy-reported outcome measure assessing quality of life for children aged 8 years and under living with a burn injury
Source: Qual Life Res. 2020 Sep 9;30(1):239–50. doi: 10.1007/s11136-020-02627-x (PMC7847857; doi:10.1007/s11136-020-02627-x)
Supplement: Supplementary file 1 — Supplementary file1 (DOCX 22 kb) [file 11136_2020_2627_MOESM1_ESM.docx]

**Appendix A: Scaling assumptions and data quality**

|  | Data quality | Scaling Assumptions | | | |
| --- | --- | --- | --- | --- | --- |
| Scale and Items | **Missing Data (%)^*^** | **Possible**  **Range** | **Actual**  **Range** | **Mean score**  **(SD)** | **CITC** |
| Social and emotional difficulties |  |  |  |  |  |
| 1.Sad | 16 | 1-5 | 1-3 | 1.63 (0.58) | 0.51 |
| 2.Easily startled | 16 | 1-5 | 1-3 | 1.23 (0.50) | 0.40 |
| 3.Anxious | 16 | 1-5 | 1-3 | 1.30 (0.54) | 0.55 |
| 4.Shouted/upset | 16 | 1-5 | 1-5 | 1.95 (1.03) | 0.71 |
| 5.Attention | 16 | 1-5 | 1-5 | 1.30 (0.62) | 0.52 |
| 6.Shouted/own way | 16 | 1-5 | 1-5 | 2.17 (1.13) | 0.68 |
| 7.Hit/harm others | 16 | 1-5 | 1-5 | 1.34 (0.72) | 0.59 |
| 8.Clingy | 16 | 1-5 | 1-5 | 1.75 (0.88) | 0.65 |
| 9.Withdrawn | 16 | 1-5 | 1-5 | 1.19 (0.52) | 0.44 |
| 10.Cried | 16 | 1-5 | 1-4 | 1.28 (0.51) | 0.27 |
| 11.Difficult play | 16 | 1-5 | 1-3 | 1.19 (0.42) | 0.49 |
| Social and emotional well-being |  |  |  |  |  |
| 1.Happy | 15 | 1-5 | 1-5 | 4.11 (0.71) | 0.68 |
| 2.Interested play | 16 | 1-5 | 1-5 | 4.29 (0.84) | 0.75 |
| 3.Confident | 15 | 1-5 | 1-5 | 4.17 (0.83) | 0.67 |
| 4.Smile/laugh | 15 | 1-5 | 1-5 | 4.30 (0.85) | 0.72 |

**^*^** Only included missing (-9999) and not N/A. Missing % calculated on total N (133).
