# Supplementary material for: The development and validation of the CARe Burn Scale: Child Form: a parent-proxy-reported outcome measure assessing quality of life for children aged 8 years and under living with a burn injury
Source: Qual Life Res. 2020 Sep 9;30(1):239–50. doi: 10.1007/s11136-020-02627-x (PMC7847857; doi:10.1007/s11136-020-02627-x)
Supplement: Supplementary file 2 — Supplementary file2 (DOCX 18 kb) [file 11136_2020_2627_MOESM2_ESM.docx]

Appendix B: A comparison of Cronbach alpha values with and without missing data

| Scale | Cronbach's alpha- all | Cronbach's alpha- no missing |
| --- | --- | --- |
| Social and Emotional Difficulties | 0.86 | 0.87 |
| Social and Emotional Well-being | 0.86 | 0.86 |
