# Supplementary material for: The development and validation of the CARe Burn Scale: Child Form: a parent-proxy-reported outcome measure assessing quality of life for children aged 8 years and under living with a burn injury
Source: Qual Life Res. 2020 Sep 9;30(1):239–50. doi: 10.1007/s11136-020-02627-x (PMC7847857; doi:10.1007/s11136-020-02627-x)
Supplement: Supplementary file 3 — Supplementary file3 (DOCX 19 kb) [file 11136_2020_2627_MOESM3_ESM.docx]

Appendix C: Stage 3 study: Spearman rank correlations between the CARe Burn Scale: Child Form and other validated health outcome psychometric measures

|  | Social and Emotional Difficulties | Social and Emotional Well-being |
| --- | --- | --- |
| *PedsQL* |  |  |
| Physical Functioning | 0.35*** | 0.21* |
| Emotional Functioning | 0.37*** | 0.21* |
| Social Functioning | 0.40*** | 0.29** |
| Psychosocial Health Summary Score | 0.42*** | 0.25** |

* P <0.05, ** P<0.01 *** P<0.001. Bonferroni adjusted.
