# Supplementary material for: The development and validation of the CARe Burn Scale: Child Form: a parent-proxy-reported outcome measure assessing quality of life for children aged 8 years and under living with a burn injury
Source: Qual Life Res. 2020 Sep 9;30(1):239–50. doi: 10.1007/s11136-020-02627-x (PMC7847857; doi:10.1007/s11136-020-02627-x)
Supplement: Supplementary file 4 — Supplementary file4 (DOCX 26 kb) [file 11136_2020_2627_MOESM4_ESM.docx]

| **Appendix D: Stage 3 study:** Fully adjusted regression coefficients and resulting P-values for demographic variables and the *Social and Emotional Difficulties* and *Social and Emotional Well-being* scales (weighted total scores). N total = 133. Significant values with P<0.05 are marked with ^*^. | | | | | | | | | | | | | | | | | | | |  | | |  |
| --- | --- | --- | --- | --- | --- | --- | --- | --- | --- | --- | --- | --- | --- | --- | --- | --- | --- | --- | --- | --- | --- | --- | --- |
| **Scales** | Child’s Age now (years) | | | Time since burn (years) | | | Child’s Gender (female) | | | | Ethnicity  (ethnic minority groups) | | | Marital status  (not married/civil partnered) | | | Cause (non-liquid) | | | Parent gender (male/female) | | | |
|  | **Coef.** | **95% CI** | **P** | **Coef.** | **95% CI** | **P** | **Coef.** | **95% CI** | **P** | | **Coef.** | **95% CI** | **P** | **Coef.** | **95% CI** | **P** | **Coef.** | **95% CI** | **P** | **Coef.** | **95% CI** | **P** | |
| Social and emotional difficulties (n=103) | 3.34 | 0.69, 5.99 | 0.01^*^ | -4.43 | -8.29, -0.56 | 0.03^*^ | -0.59 | -7.53, 8.71 | | 0.89 | 3.26 | -7.34, 13.86 | 0.54 | 5.06 | -3.66, 13.78 | 0.25 | -5.89 | -14.32, 2.54 | 0.17 | -8.43 | -20.49, 3.63 | 0.17 | |
| Social and emotional well-being  (n=106) | -0.18 | -3.86, 3.51 | 0.92 | 0.29 | -5.15, 5.74 | 0.91 | 14.22 | 2.88, 25.55 | | 0.01* | -10.74 | -25.70, 4.21 | 0.16 | 0.03 | -12.15, 12.21 | 0.99 | -7.45 | -19.16, 4.25 | 0.21 | -5.08 | -22.13, 11.97 | 0.56 | |
