# Supplementary material for: The development and validation of the CARe Burn Scale: Child Form: a parent-proxy-reported outcome measure assessing quality of life for children aged 8 years and under living with a burn injury
Source: Qual Life Res. 2020 Sep 9;30(1):239–50. doi: 10.1007/s11136-020-02627-x (PMC7847857; doi:10.1007/s11136-020-02627-x)
Supplement: Supplementary file 5 — Supplementary file5 (DOCX 26 kb) [file 11136_2020_2627_MOESM5_ESM.docx]

**Appendix E: CARe Burn Scale: Child Form (parent-proxy measure) – subscale items in each domain**

**Wound/Scar Treatments:** (scored on a 1 – 6 point Likert scale labelled: Not at all, A little, Somewhat, Quite a bit, A lot, N/A)

| *In the PAST WEEK…how bothered has your child been…* |
| --- |
| ..during wound dressing or bandage changes |
| ..when washing the body part affected by their burn wounds/scars |
| ..when wearing bandages, dressings or pressure garments (e.g. tight fitted clothing that helps control scarring) |
| ..during creaming or massage |
| ..when completing physiotherapy exercises such as stretching |
| ..about the pain/discomfort or side effects when receiving treatments for their burn (e.g. dressing/ bandage changes, creaming/ massage, physiotherapy, pressure garments, and taking medication) |

**Physical Well-being:** (scored on a 1-6 point Likert scale labelled: None of the time, A little of the time, Some of the time, Most of the time, All of the time, N/A)

| *In the PAST WEEK, how often has your child been able to…* |
| --- |
| ..do the physical activities that they wanted to do (e.g. walk/run/sit down/play) |
| ..do physical activities that other children their age/ability can do |
| ..have enough energy to do the activities they want to do |

**Wound/Scar Discomfort:** (scored on a 1 – 6 point Likert scale labelled: Not at all, A little, Somewhat, Quite a bit, A lot, N/A)

| *In the PAST WEEK, how often have your child’s burn wounds/scars been.….* |
| --- |
| ..itchy |
| ..painful |

**Social and Emotional Difficulties:** (scored on a 1-6 point Likert scale labelled: None of the time, A little of the time, Some of the time, Most of the time, All of the time, N/A)

| *During the PAST WEEK, how often has your child…..* |
| --- |
| ..been sad |
| ..got easily startled |
| ..been anxious |
| ..shouted when they were upset |
| ..found it hard to pay attention in nursery or school |
| ..shouted when they did not get their own way |
| ..hit or harmed others |
| ..been clingy in social situations |
| ..withdrawn from social situations |
| ..cried without reason |
| ..had difficulty playing with others |

**Social and Emotional Well-being:** (scored on a 1-6 point Likert scale labelled: None of the time, A little of the time, Some of the time, Most of the time, All of the time, N/A)

| *During the PAST WEEK, how often has your child been…* |
| --- |
| ..happy |
| ..interested in play activities |
| ..confident in themselves |
| ..smiled, laughed or giggled when they played |

**Usage and scoring instructions:**

After scoring, for all domains, higher scores reflect better health outcomes.

Copies of the formatted scales with instructions ready for use can be downloaded for free from [**www.careburnscales.org.uk**](http://www.careburnscales.org.uk)

Information on how to score this scale and scoring templates can be downloaded for free from [**www.careburnscales.org.uk**](http://www.careburnscales.org.uk)

*The checklists: Wound/scar treatments, Physical Well-being and Wound/scar discomfort are scored by summing the items (using 0 for N/A).
